# Mortality Risk Associated with Short-Term Exposure to Traffic Particles and Sulfates

**DOI:** 10.1289/ehp.9537

**Published:** 2007-01-29

**Authors:** Dan Maynard, Brent A. Coull, Alexandros Gryparis, Joel Schwartz

**Affiliations:** 1 Department of Biostatistics; 2 Department of Environmental Health and; 3 Department of Epidemiology, Harvard School of Public Health, Boston, MA, USA

**Keywords:** air pollution, black carbon particles, fine particles, GIS-based exposure, land use regression, mortality, particulate matter, traffic

## Abstract

**Background:**

Many studies have shown that airborne particles are associated with increased risk of death, but attention has more recently focused on the differential toxicity of particles from different sources. Geographic information system (GIS) approaches have recently been used to improve exposure assessment, particularly for traffic particles, but only for long-term exposure.

**Objectives:**

We analyzed approximately 100,000 deaths from all, cardiovascular, and respiratory causes for the years 1995–2002 using a case–crossover analysis.

**Methods:**

Estimates of exposure to traffic particles were geocoded to the address of each decedent on the day before death and control days, with these estimates derived from a GIS-based exposure model incorporating deterministic covariates, such as traffic density and meteorologic factors, and a smooth function of latitude and longitude.

**Results:**

We estimate that an IQR increase in traffic particle exposure on the day before death is associated with a 2.3% increase [95% confidence interval (CI), 1.2 to 3.4%] in all-cause mortality risk. Stroke deaths were particularly elevated (4.4%; 95% CI, −0.2 to 9.3%), as were diabetes deaths (5.7%; 95% CI, −1.7 to 13.7%). Sulfate particles are spatially homogeneous, and using a central monitor, we found that an IQR increase in sulfate levels on the day before death is associated with a 1.1% (95% CI, 0.1 to 2.0%) increase in all-cause mortality risk.

**Conclusions:**

Both traffic and powerplant particles are associated with increased deaths in Boston, with larger effects for traffic particles.

Many time-series studies have shown that ambient particulate matter (PM) pollution, generally measured as particles with aerodynamic diameter ≤ 10 μm (PM_10_), is associated with increased risk of deaths for cardiovascular or respiratory causes in both Europe and the United States (Katsouyanni et al. 1997; Samet et al. 2000; Schwartz 1991, 2000). More recent multicity studies have shown the associations generally persist unchanged after control for other air pollutants (Dominici et al. 2005), although some single-city studies have shown some sensitivity to this choice. Further, this association remains after matching on other air pollutants and temperature in case–crossover analyses (Schwartz 2004a, 2004b).

Less work has been reported using particles from specific sources, which is a critical need for regulators. Several studies have examined intermediary markers and reported that traffic particles seemed to be more toxic than others. In a case–crossover analysis of 772 patients presenting to Boston-area hospitals with strictly defined myocardial infarction, Peters et al. (2001) reported that elevated concentrations of ambient particulates were associated with higher risk. The risk was even greater if black carbon (BC), a surrogate for traffic particles, was used as the exposure. Schwartz and coworkers (2005) reported a stronger association of BC than of PM with aerodynamic diameter ≤ 2.5 μm (PM_2.5_) with heart rate variability. Less directly, Janssen and coworkers (2002) showed that the increased risk of cardiovascular hospitalization in 14 U.S. cities associated with a fixed increment in exposure to airborne particles varied with the particle composition in the city. It increased with the percent of the particles from traffic sources, and decreased with the percent of particles from windblown dust. Laden and coworkers (2000) analyzed data from the Harvard Six Cities study and found different toxicity for particles from different sources. They found that a 10-μg/m^3^ increase in ambient particles was associated with an increase in daily deaths of 3.4% for mobile source particles and 1.1% for coal combustion particles. Further, the sulfate particles were more strongly associated with respiratory deaths. However, in this study particles from both traffic and coal-burning power plants were estimated from central-site monitoring (Laden et al. 2000).

The ability of stationary air monitoring (SAM) site PM concentrations to reflect corresponding exposures depends largely on the extent of spatial heterogeneity in PM levels, which varies by particulate component. Sulfate, a major component of PM_2.5_ in Boston, is a stable particle species that varies little outdoors across wide geographic areas (Suh et al. 1995).

Studies of traffic-related particles have used tracers to reflect total traffic-related particulate emissions, of which elemental carbon (EC) and filter blackness are the most common, particularly for diesel exhaust. Both markers have been shown to vary substantially within a city, with a 4-fold range of variations across different streets in Harlem, New York, related to bus and truck counts on adjacent streets (Kinney et al. 2000). Another study (Brauer et al. 2003) showed that traffic variables explained 81% of the variability of filter blackness across 41 sites within Munich, Germany, further validating the measure as a marker of traffic particles.

This suggests that substantial exposure misclassification could result from the use of a central measuring site to assess exposure to traffic particles, and that a geographically based approach could substantially improve the assessment. We have conducted a case–crossover analysis of approximately 100,000 hospital deaths in the Boston metropolitan area over the years 1995–2002, using a spatial–temporal land use regression estimate of the daily concentrations of traffic particles at the home of each decedent and central-site readings of sulfate levels.

## Data and Methods

### Health data

Individual mortality records were obtained from the Massachusetts Department of Public Health, for the years 1995–2002. These records included information such as residential location, place of death, age, sex, date of death, ethnicity, education, and primary cause of death. Specific cause mortality was derived from the *International Classification of Diseases* (ICD) codes [*9th Revision* before 1999 (World Health Organization 1975) and *10th Revision* 1999 to 2002 World Health Organization 1993)]. Cardiovascular disease (CVD) was defined as ICD-9 390–429, and ICD-10 I01–I52. Stroke was defined as ICD-9 430–438 and ICD-10 I60–I69. Respiratory deaths were ICD-9 460–519 and ICD10 J00–J99. Diabetes was defined as ICD-9 250 or ICD-10 E10–E14.

Each individual’s residential location at time of death was geocoded (in terms of latitude and longitude) using a commercial geocoding firm (Tele Atlas, Lebanon, NH).

No BC data were available from March 1997 to April 1999, so deaths during this period were not included in the analysis. Of the remaining 192,822 deaths, we had an imprecise place of residence for 6,230 of these (a necessary variable to predict location-specific individual BC levels), so these deaths were excluded from the analysis (Table 1). In addition, we excluded inpatient deaths because these subjects were unlikely to have been at their residence the day before death. This excluded an additional 78,667 deaths, resulting in 107,925 total deaths for the final analysis.

### Stationary air monitoring

Daily measurements of BC were obtained from a measuring location at the Harvard School of Public Health (HSPH) using an aethalometer (Magee Scientific, Berkeley, CA). From March 1997 to December 1999 the HSPH pollution monitor was shutdown, so no data are available for this time period. During April–December 1999, the Massachusetts Department of Environment operated an identical monitor at a nearby site (Roxbury). We used monitoring data from that site for that period, after calibrating the measurements to the HSPH monitor using a linear regression during the period when both monitors were operating. Overall, 12.6% of the days in our study used BC data from the Roxbury monitor. Hence, our analysis focuses on mortality between 1995–1997 and 1999–2002.

Sulfate data were measured at HSPH using HEADS impactors (URG, Chapel Hill, NC), and were available from 25 September 1999 to the end of the study (31 December 2002). Weather data were obtained from the National Climatic Data Center.

### Exposure modeling

In order to predict local BC level, we used a validated spatial–temporal land use regression model to predict 24-hr measures of traffic exposure data (BC) at > 80 locations in the Boston area. Three-quarters of the sites were residential, and the rest were at commercial or government facilities. The data consist of > 6,021 BC observations from 2,127 unique exposure days. A detailed description of all exposure data sources are provided in Section 2 of the study by Gryparis et al. (2007). Predictors in the regression were the BC value at the stationary monitor (to capture average concentrations in the area on that day), meteorologic conditions and other characteristics (e.g., weekday/weekend) of a particular day, as well as measures of the amount of traffic activity [e.g., GIS (geographic information system)-based measures of cumulative traffic density within 100 m, population density, distance to nearest major roadway, percent urbanization] at a given location. Cumulative traffic density measure is recorded once per location. We used non-parametric regression methods to allow these factors to affect exposure levels in a potentially nonlinear way. Finally, we used thin-plate splines, a two-dimensional extension of nonparametric regression terms, to model longitude and latitude and capture additional spatial variability unaccounted for after including our deterministic spatial predictors in the model. This approach is a form of universal kriging (i.e., kriging extended to incorporate covariates) or a geoadditive model (Kammann and Wand 2003) for daily concentrations of particle levels. We had complete information on all these factors for 2,114 of the 2,127 unique exposure days.

Specifically, let *Y_ij_* be the log-transformed BC concentration for the *j^th^* location on day *i*. The model expresses *bc* (BC) concentration as a function day of the week, ambient levels from the monitor located at the HSPH, meteorologic conditions [*Temp* (temperature), *R.Hum* (relative humidity)], day of the year, cumulative traffic density within 100 m (*CumTraffic*), and a nonparametric function of longitude and latitude (*lat,lon*). The model is:


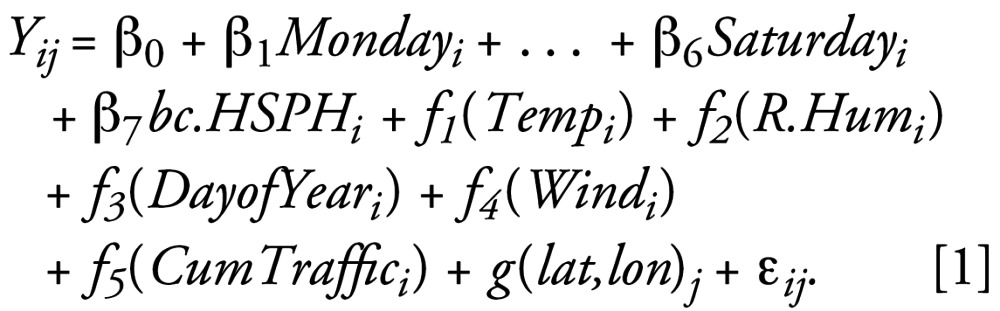


Gryparis et al. (2007) showed that the residuals obtained from the fitted model do not exhibit additional residual correlation over either time or space, indicating that the temporal and spatial components of the model adequately characterize correlations among the observed pollution. A separate model was fit for the warm (May–October) and cold (November–April) seasons. The *R*^2^ of the model (over both seasons) was 0.82, and the cross-validated *R*^2^ between the daily measurements taken outside the residential locations and corresponding predictions obtained from fitting the model to the data, excluding data from a particular residential location, was 0.36. This is in contrast to an *R*^2^ of 0.09 for the association between the central site and each residential reading. No other traffic variable was significant after controlling for cumulative traffic.

It is important to emphasize that the proposed model specifies a lack of space–time interaction on the log scale, so that the percent change over time, not the raw concentration, is constant across locations. Thus, the model implies that locations with higher levels have larger differences in concentrations over time, and these different changes in time are used in the health effects models.

### Analytical methods

We investigated the association between daily concentrations of BC and sulfate and mortality risk using a bidirectional case–crossover design (Bateson and Schwartz 1999) with time-stratified control sampling (Levy et al. 2001). By definition of the case–crossover design, each case acted as its own control on a set of predefined days (called a reference period) around the time it became a case. For a particular subject, the reference period was chosen to consist of the pollution concentrations for every third day within the same month of the same year that death occurred. For example, if a subject died on 23 November 2000, then November 2, 5, 8, 11, 14, 17, 20, 26, and 29 of 2000 were defined as controls days. Every third day was chosen to reduce autocorrelation between concentrations on successive days, while maintaining power. More details on this approach have been published previously (Zanobetti and Schwartz 2005; Zeka et al. 2006). Because a particular subject is his or her own control, the case–crossover design inherently accounts for subject-specific, time-invariant confounders. Using a month-long control period has been shown by simulation to produce unbiased estimates of effect sizes even in the presence of strong seasonal confounding (Schwartz et al. 2003).

The National Morbidity, Mortality, and Air Pollution Study (Dominici et al. 2005) indicates the strongest particle–mortality association with the previous day’s concentration, and we used that lag in our models. Thus, we report results that compare the exposure the day before each subject’s death with the exposures the subject experienced the day before control days. We express effect size estimates as the percent increase in risk of mortality associated with an interquartile range (IQR) increase in particle concentrations.

Risk may vary nonlinearly with apparent temperature (defined as an individual’s perceived air temperature given the humidity), given by the formula:


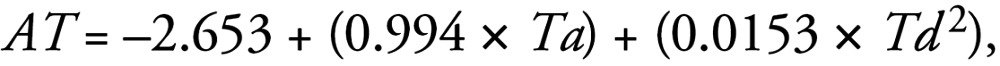


where *Ta* is air temperature and *Td* is dew point temperature (O’Neill et al. 2003). We therefore used a regression spline with 4 degrees of freedom for apparent temperature on the day of death. Apparent temperature the previous day was treated linearly because previous analyses in Boston have shown a linear relation. A 4-degree-of-freedom spline was also considered for previous day’s temperature. Day of the week was controlled using dummy variables for each day. The data were analyzed using a conditional logistic regression (PROC PHREG in SAS (SAS release 8.2; SAS Institute Inc., Cary, NC).

In addition to analyzing all-cause mortality, we ran a series of stratified analyses to investigate how the pollution–mortality association varies among different causes of death. We stratified the data according to categories of causes of death: CVD, respiratory deaths, stroke, and diabetes. We conducted the same case–crossover analysis within each stratum, in case the temperature dependence varied by cause of death.

We estimated effect modification due to factors on both the personal level and at the census tract level. Specifically, we estimated whether sex and low individual education (defined as < 12 years of education), or poverty rate and median income, defined at the census tract level, modified the association between death and particle levels. Last, to assess whether the association between BC levels and death is driven by high air pollution days, we ran a broken stick model that specified two slopes for BC levels, one below and one above the median BC level. Because sulfate data were more limited, this analysis was only done for BC.

## Results

Of the 107,925 deaths included in our analyses, 43.0% of subjects were male, 94.4% were white, and 26.1% had higher than a high school education (Table 2). The average age among these 107,925 subjects was 76.6 years.

Table 3 contains a summary of the exposure and meteorologic variables used in the analysis. Sulfate data were available only for about half as many days as data for BC. The correlation between HSPH BC levels and predicted BC levels for an individual was fairly high, approximately 0.80, whereas the correlations with apparent temperature were 0.22 and 0.05 for predicted BC and HSPH BC levels, respectively. Sulfate levels were moderately correlated with both predicted BC levels and HSPH-BC levels, at approximately 0.44 for both variables. Mean predicted BC at residences was lower than the mean BC level at the central-site monitor, reflecting its downtown location.

Table 4 shows the results from the regression analyses for all-cause mortality. We estimate that an IQR increase in previous day’s predicted BC level is associated with a 2.3% [95% confidence interval (CI), 1.2 to 3.4%] increase in all-cause mortality risk. These results were essential unchanged when we used a 4-degree-of-freedom spline for previous day’s temperature.

Table 4 also shows estimated associations between mortality and sulfate levels at the central site. We estimate that an IQR increase in sulfate level on the day before death is associated with a 1.1% (95% CI, 0.01 to 2.0%) increase in mortality risk. The sample size was reduced substantially in the sulfate analysis. In the bivariate analysis that included both sulfate and predicted BC in the model, we observed a similar effect estimate for an IQR increase BC level, at 2.2% (95% CI, 0.16 to 4.2%), although the effect of an IQR increase in sulfate levels dropped to 0.45% (95% CI, −0.45 to 1.6%). Because of limitations on when monitors were operated, this analysis contained fewer deaths than either univariate analysis.

Table 5 shows results of the stratified cause-specific analyses. We estimate the largest BC associations for respiratory deaths, stroke, and diabetes, with the association with stroke being statistically significant. Diabetes showed a particularly large increase (5.7%; 95% CI, −1.7 to 13.7%), but was based on a small number of deaths (Table 2). We estimate that the association between mortality and an IQR increase in sulfate on the day before death is largest for respiratory deaths and death by stroke, although none of these associations were statistically significant.

Because some studies have suggested that the sulfate–mortality association has a different lag than for other particles (e.g., Ito et al. 2006), we examined longer lags; however, the largest association was seen at lag 1 (results not shown).

We also estimated possible effect modification on both the personal and census tract levels. Personal variables evaluated included sex and education, whereas census tract variables considered were poverty rate and median income. None of these interaction terms were even marginally significant (*p* > 0.50).

Further, the broken stick model specifying two BC slopes defined below and above the median BC level (0.22 μg/m^3^), showed no evidence of nonlinearity of the BC slope (*p* = 0.32 for a change in slope).

## Discussion

We have shown that both traffic particles, as indexed by BC, and sulfate particles are associated with increased risk of death in Boston. The association with traffic particles derived from a GIS-based exposure assessment, which has not previously been used in examining the acute effects of particle exposure. The association with traffic particles was independent of the sulfate association. The association with sulfate was reduced, and lost significance, after control for traffic particles. The lack of significance may be attributed partly to the reduced sample size. Moreover, the effects of traffic particles were seen for respiratory, cardiovascular, and stroke deaths, whereas the sulfate associations were predominantly with respiratory deaths and strokes, with no association with cardiovascular deaths, similar to the results of Laden et al. (2000).

The finding of an association with traffic particles, especially the finding regarding vascular health, is consistent with recent acute exposure studies suggesting that traffic particles may be associated with myocardial infarctions (Peters et al. 2001, 2004), arrhythmias (Peters et al. 2000), alterations in heart rate variability (Schwartz et al. 2005), and impaired endothelial function (O’Neill et al. 2005). It is also consistent with chronic exposure studies such as that of Hoek et al. (2002), who found that relative risk of death associated with spatial variability in traffic particles was larger than the risk reported for PM_2.5_ in other cohort studies. The recent finding in the American Cancer Society study that spatial variability of PM_2.5_ exposure in Los Angeles, California, had a larger risk coefficient than for PM_2.5_ across cities is also suggestive, because traffic particles account for a larger fraction of within-city variation in concentration (Jerrett et al. 2005).

Roemer and van Wijnen (2001) examined the association with mortality indirectly, by reporting a steeper slope between ambient PM and daily deaths for persons who lived near major roads, where more of the PM was likely produced by traffic. The effect of an interquartile increase in BC in our study was larger than has previously been noted for IQR increases in PM_2.5_, again suggesting greater toxicity.

The association between sulfate particles and daily deaths in our study is consistent with the results published by Laden et al. (2000), analyzing data from two decades earlier. Indeed, given that the IQR for sulfates in our study was 2.26 μg/m^3^, the effect size we found, even after controlling for traffic particles, is about twice as big as reported in the Laden study. Hence, the lack of significance in the model adjusting for traffic particles is most likely a power issue. Other studies, using source apportionment techniques, have also reported associations with sulfate factors (e.g., Ito et al. 2006). Although their inclusion of mass other than SO_4_^2−^ per se precludes a quantitative comparison, they qualitatively support these results. These results are also consistent with findings of associations of sulfate particles in Montreal (Goldberg et al. 2001). Hence, even at current concentrations, sulfate particles may be a major public health risk. Sulfate particles in the Northeast are predominantly the result of long-range transport from coal-burning power plants. Indeed, a study comparing sulfate concentrations in Western Massachusetts, far from any local sources, and Boston found that the concentrations were essentially identical (Salmon et al. 1997). Therefore, control of sulfur emissions from such power plants remains a major public health concern.

Our findings do differ from the results of a study in Atlanta, Georgia (Klemm et al. 2004). However, sulfate particles are formed by photochemical processes, resulting in much higher concentrations in the summer than other periods. Studies have shown that low ventilation conditions, such as those found when central air conditioning is operating, substantially reduce both the correlation of indoor with outdoor particles and the slope of the association (e.g., Sarnat et al. 2000). Hence, ambient sulfate particle concentrations in Atlanta (the prevalence of central air conditioning was 87% in 1999) are likely a much poorer surrogate for exposure to sulfate than in Boston, where central air conditioning prevalence was 17% in 1999. Thus these differences in results could result from greater exposure error in Atlanta.

Developing data also suggest that urban sulfate particles, which are not simply ammonium bisulfate, are associated with adverse responses. For example, a recent study in Boston examined endothelial function in diabetics, as assayed using flow mediated dilation of the brachial artery (O’Neill et al. 2005). The association with sulfate particles was as strong as (and more significant than) with BC in that study. Another study reported an association between sulfates and ventricular arrhythmias in subjects with implanted defibrillators (Dockery et al. 2005).

The two-pollutant model we fit to estimate the independent effects of BC and sulfate requires some caution. First, the degree of measurement error in the two exposures may well be different, which can distort the association of collinear exposures. For instance, Zeger et al. (2000) showed that upward bias can occur in multipollutant models, but only when there is strong correlation among the pollutants or among the multiple measurement errors. In our case the correlation was modest, suggesting bias toward the null. In addition, the reduced power in such models makes chance a plausible explanatory factor for differences from the single pollutant models, particularly in a single-city analysis.

The finding of an imprecise but considerably larger association with diabetes deaths is consistent with previous studies showing that diabetics are a susceptible subgroup (e.g., Goldberg et al. 2001), as well as recent studies of deaths from diabetes (e.g., Ostro et al. 2006).

Previous studies using GIS-based land use regressions to estimate exposures have focused on estimating long-term exposure, and studies of health effects associated with such exposures have focused on longer-term effects. Often, the GIS models have been deterministic (e.g., depending on predictors such as traffic density), based on dispersion models, or based on empirical smoothing of observed data. Recently some models have tried to incorporate at least two of these approaches, and we have used land use regression predictors plus smoothing.

Our model differs in that it seeks to capture short-term temporal variations, using concentrations at a central site and weather parameters as predictors. Spatial variability in the temporal effects are captured by fitting separate spatial models for warm and cold season, where differences in average mixing heights produce different spatial patterns, and by using a log-linear model, with weather parameters predicting different percent changes in concentrations at different locations, rather than different absolute changes. This allowed us to explain 67% of the temporal variation at the 80 different locations for which we had multiday measurements of BC. Using this approach, we have captured some additional variation in exposure between event and control days that would not have been possible using a central-site monitor for each subject. We believe this represents an advance in exposure assessment for short-term effects.

The size of the estimate for traffic particles deserves some comment. Because BC is a proxy for traffic particles, the true fine mass concentration (in micrograms per cubic meter) of traffic particles in Boston is greater than the concentration of BC. This is one major reason we have focused on the IQR rather than a specific increment (the other is for comparison with sulfates). However, the size of effect for an IQR change in exposure is large compared with other studies of the acute effects of PM on mortality. This is likely attributed to a combination of less measurement error (compared with central-site measurements), reducing the downward bias produced by measurement error, and greater toxicity of traffic particles than particles from other sources.

A potential statistical issue that arises when using spatial–temporal predictions of exposure is that these quantities are uncertain rather than measured quantities. Thus, there is some uncertainty associated with these measures, which could represent a different type of measurement error that could bias the resulting health effect estimates. We have begun to investigate this issue, and preliminary simulation studies suggest that in logistic regression settings in which the true effect is relatively small, this bias is neglible. Such empirical findings agree with the bias formulas for logistic regression in the work of Carroll et al. (1995).

In sum, we find evidence that both traffic particles and particles from coal burning power plants are associated with increased risk of mortality in the Boston metropolitan area, with the traffic particle association more significant, and larger. Because Boston currently is in compliance with the current and proposed U.S. Environmental Protection Agency (2006) PM_2.5_ standard, this suggests that current standards are not protective of public health.

## Figures and Tables

**Table 1:** Deaths in the Boston metropolitan area 1995–1997 and 1999–2002 by exclusion criteria.

Criteria	No.
Total cases (deaths)	230,506
Missing BC data	37,684
Inpatient deaths	78,667
Imprecise residence location	6,230
Total restricted cases	107,925

**Table 2:** Descriptive statistics: deaths out of hospital in Boston metropolitan area, 1995–2002.

Characteristic	No. (%)
Sex	
Male	46,377 (43)
Female	61,548 (57)
Race	
White	101,976 (94.4)
Black	4,164 (3.9)
Other	1,785 (1.7)
Education (years)	
0–12	78,496 (72.7)
> 12	28,181 (26.1)
Missing	1,248 (1.2)
Cause of death	
CVD	33,785 (31.3)
Stroke	6,070 (5.6)
Respiratory	9,878 (9.2)
Diabetes	2,694 (2.5)
Other	55,498 (51.4)

**Table 3:** Descriptive statistics for exposure and meteorologic covariates, Boston 1995–2002.

Covariate	Mean	Median	SD	Range	IQR	Q1	Q3	Days of data available
Apparent temp (°C)	9.715	8.232	10.315	45.793	17.724	0.849	18.573	2,705
HSPH BC (g/m)	1.059	0.897	0.679	7.188	0.757	0.591	1.348	2,127
GIS-based BC (g/m)	0.255	0.218	0.171	2.619	0.203	0.132	0.334	2,114
HSPH sulfate	3.087	2.378	2.514	28.958	2.259	1.531	3.79	1,136

temp, temperature. Q1 and Q3 are quartiles.

**Table 4:** Percent increase in deaths for IQR increase in exposure, Boston 1995–2002.

Exposure measure	*p*-Value	Percent increase for IQR increase (95% CI)	No. of cases in analysis
Univariate			
GIS-based BC	< 0.0001	2.3 (1.2 to 3.4)	107,925
HSPH sulfate	0.0169	1.1 (0.01 to 2.0)	64,080
Bivariate			
GIS-based BC	0.0339	2.2 (0.16 to 4.2)	57,029
HSPH sulfate	0.2991	0.45 (−0.45 to 1.6)	

**Table 5:** Stratified cause of death-specific regression results (lag-1).

Cause of death	Particle type	*p*-Value	Percent increase for IQR increase (95% CI)
CVD	BC	0.13	1.5 (−0.4 to 3.4)
	Sulfate	0.72	−0.2 (−1.5 to 1.0)
Stroke	BC	0.06	4.4 (−0.2 to 9.3)
	Sulfate	0.39	2.0 (−2.4 to 6.1)
Respiratory	BC	0.04	3.7 (0.1 to 7.4)
	Sulfate	0.20	2.1 (−1.1 to 5.3)
Diabetes	BC	0.13	5.7 (−1.7 to 13.7)
	Sulfate	0.36	2.9 (−3.1 to 9.5)
